# Intra-bin correction and inter-bin compensation of respiratory motion in free-running five-dimensional whole-heart magnetic resonance imaging

**DOI:** 10.1016/j.jocmr.2024.101037

**Published:** 2024-03-16

**Authors:** Christopher W. Roy, Bastien Milani, Jérôme Yerly, Salim Si-Mohamed, Ludovica Romanin, Aurélien Bustin, Estelle Tenisch, Tobias Rutz, Milan Prsa, Matthias Stuber

**Affiliations:** a Department of Radiology, Lausanne University Hospital and University of Lausanne, Lausanne, Switzerland; b Center for Biomedical Imaging (CIBM), Lausanne, Switzerland; c University Lyon, INSA-Lyon, University Claude Bernard Lyon 1, UJM-Saint Etienne, CNRS, Inserm, CREATIS UMR 5220, U1206, F-69621, 7 Avenue Jean Capelle O, 69100 Villeurbanne, France; d Department of Radiology, Louis Pradel Hospital, Hospices Civils de Lyon, 59 Boulevard Pinel, 69500 Bron, France; e Advanced Clinical Imaging Technology, Siemens Healthineers International AG, Lausanne, Switzerland; f IHU LIRYC, Electrophysiology and Heart Modeling Institute, Université de Bordeaux – INSERM U1045, Avenue du Haut Lévêque, 33604 Pessac, France; g Department of Cardiovascular Imaging, Hôpital Cardiologique du Haut-Lévêque, CHU de Bordeaux, Avenue de Magellan, 33604 Pessac, France; h Service of Cardiology, Heart and Vessel Department, Lausanne University Hospital and University of Lausanne, Lausanne, Switzerland; i Division of Pediatric Cardiology, Woman-Mother-Child Department, Lausanne University Hospital and University of Lausanne, Lausanne, Switzerland

**Keywords:** Whole heart magnetic resonance imaging, Motion correction, Motion compensation, Free-breathing, Free-running

## Abstract

**Background:**

Free-running cardiac and respiratory motion-resolved whole-heart five-dimensional (5D) cardiovascular magnetic resonance (CMR) can reduce scan planning and provide a means of evaluating respiratory-driven changes in clinical parameters of interest. However, respiratory-resolved imaging can be limited by user-defined parameters which create trade-offs between residual artifact and motion blur. In this work, we develop and validate strategies for both *correction* of intra-bin and *compensation* of inter-bin respiratory motion to improve the quality of 5D CMR.

**Methods:**

Each component of the reconstruction framework was systematically validated and compared to the previously established 5D approach using simulated free-running data (N = 50) and a cohort of 32 patients with congenital heart disease. The impact of intra-bin respiratory motion *correction* was evaluated in terms of image sharpness while inter-bin respiratory motion *compensation* was evaluated in terms of reconstruction error, compression of respiratory motion, and image sharpness. The full reconstruction framework (intra-acquisition correction and inter-acquisition compensation of respiratory motion [IIMC] 5D) was evaluated in terms of image sharpness and scoring of image quality by expert reviewers.

**Results:**

Intra-bin motion *correction* provides significantly (p < 0.001) sharper images for both simulated and patient data. Inter-bin motion *compensation* results in significant (p < 0.001) lower reconstruction error, lower motion compression, and higher sharpness in both simulated (10/11) and patient (9/11) data. The combined framework resulted in significantly (p < 0.001) sharper IIMC 5D reconstructions (End-expiration (End-Exp): 0.45 ± 0.09, End-inspiration (End-Ins): 0.46 ± 0.10) relative to the previously established 5D implementation (End-Exp: 0.43 ± 0.08, End-Ins: 0.39 ± 0.09). Similarly, image scoring by three expert reviewers was significantly (p < 0.001) higher using IIMC 5D (End-Exp: 3.39 ± 0.44, End-Ins: 3.32 ± 0.45) relative to 5D images (End-Exp: 3.02 ± 0.54, End-Ins: 2.45 ± 0.52).

**Conclusion:**

The proposed IIMC reconstruction significantly improves the quality of 5D whole-heart MRI. This may be exploited for higher resolution or abbreviated scanning. Further investigation of the diagnostic impact of this framework and comparison to gold standards is needed to understand its full clinical utility, including exploration of respiratory-driven changes in physiological measurements of interest.

## Graphical abstract

**Figure:**
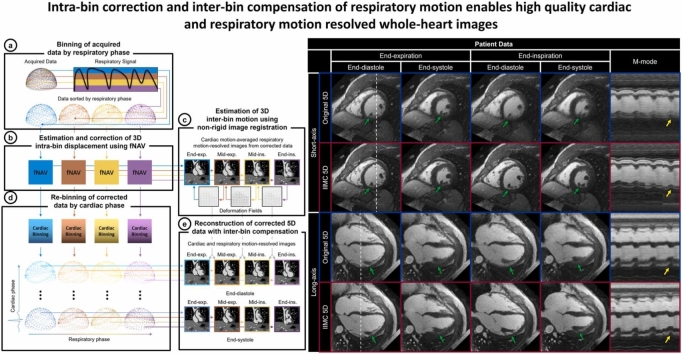

## 1 Background

Three-dimensional (3D) time-resolved (CINE) whole-heart magnetic resonance imaging (MRI) is increasingly used to assess anatomical structures and ventricular function in diseases that affect the cardiovascular system [1], [2], [3], [4]. When compared to conventional two-dimensional (2D) CINE techniques, 3D acquisitions with isotropic spatial resolution can drastically reduce the complexity of prospective scan planning by enabling targeted and retrospective reformatting of images to interrogate the complex cardiac anatomy [4], [5] after the scan. Nevertheless, the acquisition time for most 3D sequences published to date precludes conventional breath-holding and therefore respiratory motion compensation is required to obtain diagnostically useful images. This may be achieved using hardware-based or image-based [6], [7] navigators that limit the data acquisition to a predetermined respiratory phase (i.e., gating to end-expiration [End-Exp]), using navigators or intermediate image reconstructions to correct motion [8], [9], [10], [11], [12], [13], [14], or by binning and reconstructing the acquired data into respiratory motion-resolved images using compressed sensing (CS) [15], [16].

Respiratory motion-resolved imaging, by virtue of scanning throughout the entire respiratory cycle, improves acquisition efficiency, which may in turn lead to increased resolution or decreased total scan time. It also provides a means of evaluating respiratory-driven changes in clinical parameters of interest, such as cardiac output [17] or blood flow [18]. However, two main limitations exist, which are related to user-defined parameters. First, the number of reconstructed respiratory bins presents a trade-off between the level of undersampling, which may lead to residual artifact, and the amount of remaining motion within each reconstructed bin (intra-bin), which may lead to image blur. Second, a weighting parameter in the CS reconstruction balances data consistency and regularization along the respiratory dimension and must be chosen such that undersampling artifact is reduced without introducing blur due to compression of the remaining motion between each reconstructed bin (inter-bin). As a result of both of these factors, image quality is to some degree user-dependent, and both the achievable resolution and acceleration factor for respiratory-resolved imaging may be constrained.

In this work, we develop and validate strategies for both intra-bin *correction* and inter-bin *compensation* of respiratory motion to improve the quality of previously established free-running cardiac and respiratory motion-resolved five-dimensional (5D) CMR reconstructions in terms of sharpness, residual artifact level, and fidelity of the underlying respiratory motion [19], [20], [21]. We validate our approach using a comprehensive numerical simulation and systematically compare reconstructions with and without the proposed motion *correction* and *compensation* in a cohort of 32 patients with congenital heart disease (CHD).

We test the following three hypotheses. 1) *Correction* of intra-bin motion using focused navigation (fNAV) will improve image sharpness relative to uncorrected images [22]. 2) The inclusion of non-rigid deformation fields for inter-bin *compensation* in the CS reconstruction will enable larger regularization weights to reduce artifact without incurring motion blur [23], [24], [25]. 3) The combined approach for intra-bin *correction* and inter-bin *compensation* of respiratory motion will result in improved image quality for 5D whole-heart MRI across the reconstructed respiratory bins relative to the previously established approach for 5D imaging [19], [20], [21].

## 2 Methods

### 2.1 Data acquisition

**Fig. 1:**
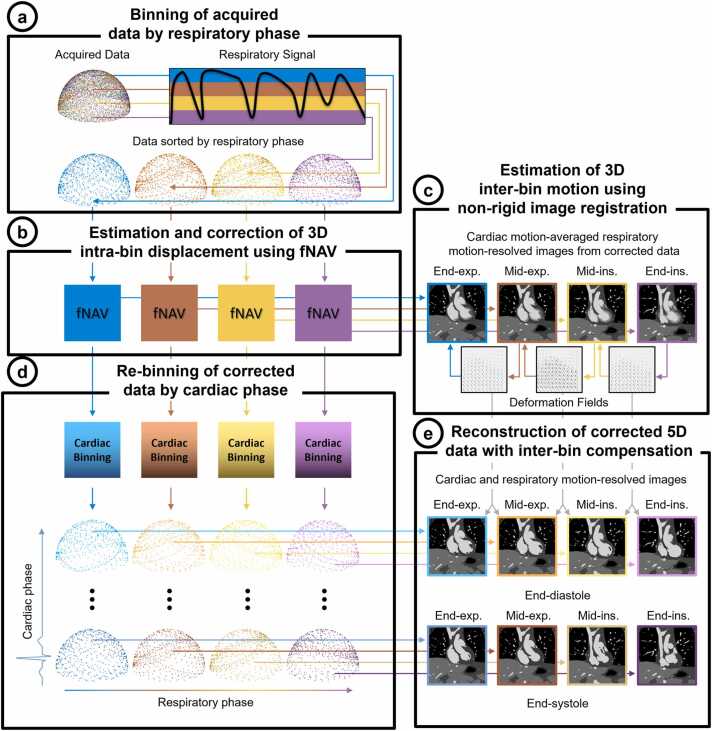
Schematic overview of the proposed framework for cardiac and respiratory motion-resolved 5D whole-heart imaging with intra-bin *correction* and inter-bin *compensation* of respiratory motion. Free-running data are sorted into respiratory bins (a) and bulk translational displacement of the heart due to respiration motion is estimated within each bin using fNAV (b). The translational displacement estimates from (b) are used to correct the data within each respiratory bin and an intermediate respiratory motion-resolved reconstruction where cardiac motion is averaged within each bin is used to estimate non-rigid motion between bins (c). The data within each respiratory bin that has been corrected by fNAV are further sorted into cardiac phases (d). The deformation fields estimated in (c) are integrated into a CS algorithm to reconstruct the binned data (d) and produce cardiac and respiratory motion-resolved 5D images with *correction* of intra-bin and *compensation* of inter-bin respiratory motion (e). 3D: three-dimensional, 5D: five-dimensional, End-Exp: end-expiration, End-Ins: end-inspiration, fNAV: focused navigation, Mid-Exp: mid-expiration, Mid-Ins: mid-inspiration.

Free-running data [20], [21], [26] with spiral phyllotaxis radial sampling [27] are acquired continuously and for a fixed scan time, without electrocardiogram (ECG)-triggering or respiratory navigators and therefore independent from the underlying cardiac and respiratory motion (Fig. 1a). At the beginning of each radial interleave, a readout oriented along the superior-inferior direction is acquired for subsequent extraction of both cardiac and respiratory self-gating signals using principal component analysis [20].

### 2.2 Reconstruction framework

The proposed reconstruction framework is outlined in Fig. 1 with additional details provided in the sections below. Briefly, free-running data are initially sorted into respiratory bins with cardiac motion averaged within each bin (Fig. 1a). Bulk translational displacement of the heart due to respiratory motion is estimated within each bin using fNAV (Fig. 1b). These translational displacement estimates are used to correct the original k-space data (intra-bin *correction*). Intermediate respiratory motion-resolved images are reconstructed using standard gridding, and non-rigid respiratory motion between each bin is estimated for use in a subsequent step (Fig. 1c). The intra-bin corrected data are further sorted into cardiac phases using self-gating (Fig. 1d). Finally, a multi-dimensional CS algorithm that integrates the estimates of non-rigid respiratory motion between bins (inter-bin *compensation*) is used to reconstruct cardiac and respiratory motion-resolved 5D images with *correction* of intra-bin and *compensation* of inter-bin respiratory motion (Fig. 1e).

#### 2.2.1 Step A: Binning of acquired data by respiratory phase

The acquired free-running data are binned into respiratory phases according to the amplitude of the respiratory signal (Fig. 1a) for subsequent estimation, *correction*, and *compensation* of respiratory motion as described in the proceeding sections. The amplitude of the respiratory signal is equally divided according to the number of desired bins resulting in a variable degree of intra-bin motion which is corrected by the following step.

#### 2.2.2 Step B: Estimation and correction of 3D intra-bin displacement using fNAV

3D translational displacement of the heart due to respiration is estimated within each bin using fNAV (Fig. 1b) adapted for use with free-running data as follows [22], [28]. A respiratory self-gating signal (S) which spans each acquired time-point (t) coinciding with a given respiratory bin (r) is normalized and multiplied by three initially unknown fNAV coefficients **A**_**r**_ = [$A_{r}^{x},A_{r}^{y},A_{r}^{z}$] that describe the 3D amplitude of respiratory motion within each bin in millimeters. Using these coefficients, the k-space data within each respiratory bin can be corrected for translational motion:

(1) $${\overset{®}{y}}_{r}=y(t_{r})e^{i2\pi k(t_{r})∙A_{r}∙S\left(t_{r}\right)}$$

where (y_r_) is the original and ${\overset{®}{y}}_{r}$ is the corrected 3D radial k-space data with coordinates **k(t)** = [kx(t), ky(t), kz(t)]. Beginning with an initial estimate **A**_**r**_ = [0, 0, 0], an intermediate image is reconstructed by applying the non-uniform Fourier transform (NUFFT) to Eq. (1), and an image metric, the entropy of the gradient image [22], [29], [30], is used to quantify the amount of blur present in the image and in turn to iteratively improve the estimate of **A**_**r**_. The image metric is calculated over a region of interest containing the heart. The value of **A**_**r**_ that minimizes the image metric is solved using a steepest descent algorithm where the gradient of the image metric as a function of **A**_**r**_ is approximated numerically. A golden-section search is used to determine the optimum search step size along the gradient direction.

Once the optimum coefficients are determined, the data within each respiratory bin (Fig. 1a) are corrected and used in the subsequent reconstruction steps.

#### 2.2.3 Step C: Estimation of 3D inter-bin motion using non-rigid image registration

Using the data that are now corrected for intra-bin respiratory motion (Fig. 1b), respiratory motion-resolved images are reconstructed with cardiac motion-averaged within each bin (Fig. 1c) resulting in 1/4 of the acquired data within each bin. A non-rigid image registration algorithm is used [31], [32] to derive the 3D deformation of the heart due to respiratory motion [31], [32] between adjacent bins (Fig. 1c). The resulting deformation fields will be used to provide inter-bin *compensation* of respiratory motion and improve the CS reconstruction needed for cardiac and respiratory-resolved images as described in subsequent steps [16], [23], [24], [25].

#### 2.2.4 Step D: Re-binning of corrected data by cardiac phase

Following estimation of both intra-bin and inter-bin respiratory motion, the data, which are binned by respiratory phase (Fig. 1 a) and corrected for intra-bin motion (Fig. 1c), are further binned according to cardiac phase (Fig. 1d) using a cardiac self-gating signal as described above [20] and resulting in 1%–2% of the acquired data within each bin. These cardiac and respiratory resolved data are then reconstructed using CS, now using the deformation fields for inter-bin motion *compensation* estimated in the previous step as follows.

#### 2.2.5 Step E: Reconstruction of corrected 5D data with inter-bin compensation

Consider the following optimization problem where cardiac (c) and respiratory (r) motion-resolved images (m) with spatial and temporal dimensions (x, y, z, c, r) are reconstructed from the k-space data sorted into cardiac and respiratory bins ($\overset{®}{y})$ wherein intra-acquisition respiratory motion is corrected using fNAV (Fig. 1b). Images are reconstructed using an operator (F) that contains coil sensitivities and the NUFFT, and with total variation-based regularization along the cardiac ($\nabla_{c})$ and respiratory ($\nabla_{r})$ dimensions [15], [33].

(2) $$\hat{m}=\underset{m}{\arg\min}{\left\|\mathrm{Fm}-\overset{®}{y}\right\|}_{2}^{2}+\lambda_{r}{\left\|\nabla_{r}m\right\|}_{1}+\lambda_{c}{\left\|\nabla_{c}m\right\|}_{1}$$

The finite differences operator applied along the respiratory dimension ($\nabla_{r})$ can be written as follows for an example case of four respiratory bins.

(3) $$\nabla_{r}=\left|\begin{array}{cccc} -1 & 1 & 0 & 0\\ 0 & -1 & 1 & 0\\ 0 & 0 & -1 & 1\\ 0 & 0 & 0 & 0 \end{array}\right|$$

If we apply this operator to our image, we have the following subtractions between adjacent frames.

(4) $$\nabla_{r}∙\left[\begin{array}{c} m_{x,y,z,c,1}\\ m_{x,y,z,c,2}\\ m_{x,y,z,c,3}\\ m_{x,y,z,c,4} \end{array}\right]=\left[\begin{array}{c} m_{x,y,z,c,2}-m_{x,y,z,c,1}\\ {m_{x,y,z,c,3}-m}_{x,y,z,c,2}\\ {m_{x,y,z,c,4}-m}_{x,y,z,c,3}\\ 0 \end{array}\right]$$

To further sparsity the finite difference domain and better separate undersampling artifact from the underlying inter-bin physiological motion, we can define a new operator ($\nabla_{r}^{f})$:

(5) $$\nabla_{r}^{f}=\left|\begin{array}{cccc} -T_{1\to 2} & 1 & 0 & 0\\ 0 & -T_{2\to 3} & 1 & 0\\ 0 & 0 & -T_{3\to 4} & 1\\ 0 & 0 & 0 & 0 \end{array}\right|$$

where $T_{n\to n+1}$ is an interpolation matrix containing the non-rigid deformation resulting from the registration of m_n_ to m_n+1_. Applying this operator to our image then yields the following:

(6) $$\nabla_{r}^{f}∙\left[\begin{array}{c} m_{x,y,z,c,1}\\ m_{x,y,z,c,2}\\ m_{x,y,z,c,3}\\ m_{x,y,z,c,4} \end{array}\right]=\left[\begin{array}{c} m_{x,y,z,c,2}-{T_{1\to 2}∙m}_{x,y,z,c,1}\\ {m_{x,y,z,c,3}-T_{2\to 3}∙m}_{x,y,z,c,2}\\ {m_{x,y,z,c,4}-T_{3\to 4}∙m}_{x,y,z,c,3}\\ 0 \end{array}\right]$$

In principle, if the estimation of inter-bin respiratory motion is perfect, the remaining differences will be due to undersampling and noise which can then be removed by a CS reconstruction [33], [34]. However, the finite difference operator as defined above is asymmetric which may create uneven regularization across the respiratory bins. Therefore, we also define a second operator ($\nabla_{r}^{b}$) and apply it to our images to improve the symmetry of the regularization terms [23], [35]:

(7) $$\nabla_{r}^{b}=\left|\begin{array}{cccc} 0 & 0 & 0 & 0\\ 1 & -T_{2\to 1} & 0 & 0\\ 0 & 1 & -T_{3\to 2} & 0\\ 0 & 0 & 1 & -T_{4\to 3} \end{array}\right|$$

which can then be applied to our image as follows:

(8) $$\nabla_{r}^{b}∙\left[\begin{array}{c} m_{x,y,z,c,1}\\ m_{x,y,z,c,2}\\ m_{x,y,z,c,3}\\ m_{x,y,z,c,4} \end{array}\right]=\left[\begin{array}{c} 0\\ {m_{x,y,z,c,1}-T_{2\to 1}∙m}_{x,y,z,c,2}\\ {m_{x,y,z,c,2}-T_{3\to 2}∙m}_{x,y,z,c,3}\\ {m_{x,y,z,c,3}-T_{3\to 4}∙m}_{x,y,z,c,4} \end{array}\right]$$

Note that the deformation fields for the forward and backward operators are estimated from separate image registrations.

Therefore, in the final step of our framework (Fig. 1e), we reconstruct our cardiac and respiratory binned data that have been corrected for intra-bin respiratory motion (Fig. 1d) by solving the following optimization that integrates the estimated deformation fields (Fig. 1c), to reconstruct intra-bin corrected and inter-bin compensated (IIMC) 5D images (m):

(9) $$\hat{m}=\underset{m}{\arg\min}{\left\|\mathrm{Fm}-\overset{®}{y}\right\|}_{2}^{2}+\lambda_{r}{\left\|\nabla_{r}^{f}m\right\|}_{1}+\lambda_{r}{\left\|\nabla_{r}^{b}m\right\|}_{1}+\lambda_{c}{\left\|\nabla_{c}m\right\|}_{1}$$

which includes both the respiratory motion-compensated finite difference operators described as well as a cyclic finite difference operator [20] along the cardiac dimension ($\nabla_{c})$.

### 2.3 Study data

#### 2.3.1 Numerical simulation data

To validate the reconstruction framework outlined in the previous sections, a numerical simulation of free-running 3D radial data was developed for this work based on the Magnetic Resonance extended Cardiac-Torso (MRXCAT) [36] and extended Cardiac-Torso Cardiovascular Magnetic Resonance (XCMR) [22], [37] approaches. In summary, high-resolution (1 mm^3^) 3D volumes covering the chest were derived from the XCAT software which contains labels for each tissue of interest and produces realistic nonrigid cardiac and respiratory motion [38]. A total of 1500 unique volumes were generated from XCAT and arranged into a 5D array representing a 3D volume sampled across 50 phases of a full cardiac cycle and 30 amplitudes of respiratory motion. To mimic realistic physiological motion, cardiac cycles with heart-rate variability and respiratory cycles with variability in both frequency and amplitude were generated [37], spanning the length of the synthetic MRI acquisition with user-defined scan parameters chosen to match patient data as described below.

To generate a desired readout, a 3D XCAT volume representing the cardiac and respiratory phase for the corresponding timepoint was interpolated from the 5D array described above, and the labeled tissues were converted to MRI contrast using an analytical signal equation with relaxation values from the literature. An inverse NUFFT containing the desired radial phyllotaxis trajectory and simulated 3D coil sensitivities was then used to produce synthetic k-space data with added complex Gaussian noise to reach a user-defined signal-to-noise ratio [37]. Using this numerical simulation framework, free-running data were generated with and without cardiac motion to simplify the analyses described in the following sections. A total of 50 datasets with variable heart rate (50–90 beats per minute; bpm) and maximum respiratory motion amplitude (A) varying along all three spatial dimensions (x, y, z) from (Ax = Ay = Az = 0 mm) to (Ax = 2, Ay = 12, Az = 20 mm) were generated.

#### 2.3.2 Patient data

To demonstrate the feasibility of our approach in vivo, 32 patients with CHD (average age: 21 years, range: 1–60 years, 21 males) and a clinical indication for CMR were included in this institutional review board-approved study. Following the protocol at our institution, examinations were performed without sedation for patients older than 5 years of age (N = 30) and using intravenous dexmedetomidine (N = 2) otherwise, during free breathing, on a 1.5T clinical MRI system (MAGNETOM Sola, Siemens Healthcare, Erlangen, Germany) after administration of 2–5 mg/kg of ferumoxytol (Takeda, Tokyo, Japan) [39]. A slab-selective spoiled gradient echo prototype free-running 3D radial sequence was used [20], [26] and resulted in uninterrupted acquisitions of 6-minute duration [21]. Main sequence parameters were radiofrequency excitation angle: 15°, resolution: 1.15 mm^3^, field-of-view (FOV): 220 mm^3^, echo time/repetition time: 1.53/2.84 ms, readout bandwidth: 1002 Hz/pixel. A standard multi-slice 2D CINE balanced steady-state free precession (bSSFP) in short-axis orientation was acquired for all patients and the sequence parameters were radiofrequency excitation angle: 63°, resolution: 1.4 × 1.4 mm^2^, slice thickness: 5–8 mm, FOV: 168 × 208 mm^2^, echo time/repetition time: 1.2/2.4 ms, reconstructed cardiac phases: 25.

### 2.4 Experiments

To validate our proposed framework and explore its boundary conditions, three experiments were designed in line with our three hypotheses to assess the cumulative impact of intra-bin motion *correction*, inter-bin motion *compensation*, and finally the entire framework for IIMC 5D imaging.

#### 2.4.1 Experiment 1: Impact of intra-bin respiratory motion correction

To assess the effects of intra-bin respiratory motion *correction*, all simulated and patient datasets were reconstructed as respiratory motion-resolved four-dimensional (4D) (x, y, z, respiration) images [15] with (Corrected 4D) and without (Original 4D) intra-bin *correction* as described above (Fig. 1b). In each case, data were distributed into four bins according to the respiratory self-gating signal and reconstructed using a NUFFT. We refer to these four bins throughout the proceeding analysis as End-Exp, mid-expiration (Mid-Exp), mid-inspiration (Mid-Ins), and end-inspiration (End-Ins). Within each respiratory bin, cardiac motion was averaged, resulting in ∼1/4 Nyquist sampling per respiratory bin. CS, therefore, was omitted at this stage to decouple the effects of the intra-bin *correction* and potential blur due to regularization. For all simulated datasets the fNAV coefficients [$A_{r}^{x},A_{r}^{y},A_{r}^{z}$] used to correct intra-bin motion as described above in step B were compared to the corresponding ground truth values [${\overset{®}{A}}_{r}^{x},{\overset{®}{A}}_{r}^{y},{\overset{®}{A}}_{r}^{z}$]. For a given dataset, we calculated the resulting average intra-bin error (E_Intra_) as follows:

$$E_{\mathrm{intra}}=\frac{1}{N_{r}}\sum_{r}^{\mathrm{Nr}}\sqrt{{\left(A_{r}^{x}-{\overset{®}{A}}_{r}^{x}\right)}^{2}+{\left(A_{r}^{y}-{\overset{®}{A}}_{r}^{y}\right)}^{2}+{\left(A_{r}^{z}-{\overset{®}{A}}_{r}^{z}\right)}^{2}}\;$$

where the total error in the coefficients is averaged across the total number (N_r_) of respiratory bins (r). Additionally, the range of intra-bin displacement calculated for each respiratory bin was recorded for both simulated (ground truth values) and patient data (values measured by fNAV).

The resulting gridded 4D reconstructions with and without intra-bin *correction* were visually compared and the sharpness for each reconstruction was quantified by the average slope of sigmoid functions fitted to 10 points along the lung-liver interface boundary where residual respiratory motion is most likely to degrade image quality [40]. The 10 points were chosen close to the heart and within the region of interest used for fNAV calculation as described above. The sharpness measurements were performed and compared for all four reconstructed respiratory bins.

#### 2.4.2 Experiment 2: Impact of inter-bin respiratory motion compensation

For all simulated datasets, the estimated inter-bin respiratory deformation fields were compared to ground truth, and the error in total displacement (E_inter_) was calculated for each dataset. To assess the effects of inter-bin respiratory motion *compensation*, 4D (x, y, z, respiration) images from all simulated and patient datasets were again reconstructed with respiratory intra-bin *correction*, but now using CS with (Compensated 4D) and without (Original 4D) inter-bin motion *compensation* (Fig. 1c). Here, only data from the end-diastolic cardiac phase were included resulting in ∼2% Nyquist sampling per respiratory bin and allowing us to evaluate the effects of undersampling. For each dataset, the regularization weights ($\lambda_{r})$ were varied between 0 and 0.25 in steps of 0.025 resulting in 22 reconstructions per patient or simulated dataset. To evaluate the trade-off between undersampling artifact and motion blur, both 4D reconstructions with and without motion *compensation* across the tested $\lambda_{r}$ values were assessed in terms of reconstruction error, motion compression, and sharpness as follows.

In addition to visually comparing the two reconstruction methods, the residual artifact level was assessed by the root-mean-squared reconstruction error calculated over a region of interest containing the lung-liver interface. For simulated data, reconstruction error was calculated relative to a ground truth fully sampled reference. In the absence of a ground truth reference, reconstruction error for patient data is measured relative to 4D images with ∼1/4 Nyquist sampling and no CS as described in experiment 1.

Compression of respiratory motion due to regularization was evaluated by translational registration of the first (End-Exp) and last (End-Ins) frames of the 4D reconstructions over a region of interest containing the liver. For simulated data, motion compression is presented as the difference between the estimated and ground truth displacement in millimeters. In the absence of a ground truth reference, motion compression for patient data is measured relative to 4D images without CS as described for reconstruction error. Finally, sharpness along the lung-liver boundary was quantified in the same manner as experiment 1 but averaged across the four respiratory bins to simplify comparison versus $\lambda_{r}$.

#### 2.4.3 Experiment 3: Evaluating intra-bin corrected inter-bin compensated 5D whole-heart MRI

To evaluate the entire reconstruction framework, all simulated and patient datasets were reconstructed as cardiac and respiratory motion-resolved 5D (x, y, z, cardiac, respiration) images (Eq. (2)) with the proposed combination of intra-bin motion *correction* and inter-bin motion *compensation* (IIMC 5D), and compared to the previously established reconstruction method for these data without *correction* or *compensation* (Original 5D) [20], [21]. Here, we once again use four respiratory bins but now include 15–25 cardiac phases (Fig. 1d) depending on the simulated (mean RR-Interval: 933 ± 101 ms) or patient’s (mean RR-Interval: 926 ± 205 ms) heart rate, which ensured adequate temporal resolution (∼50 ms) to image end-systole [1], [21]. After normalizing the raw data signal by the maximum of the gridded reconstructions, images were reconstructed using the optimized respiratory regularization weights determined in experiment 2 and a cardiac regularization weight of 0.01.

Image reconstructions with and without IIMC were visually compared. Sharpness along the blood-myocardium interface was measured in all simulated and patient 5D image reconstructions by creating short-axis reformats from each reconstruction, manually selecting a slice during end-systole at the mid-ventricular level, placing points along the boundary, and measuring the average slope of sigmoid functions fitted to the interface similar to experiments 1 and 2 [40]. For both 5D image reconstruction methods, sharpness measurements were performed in the end-expiratory and end-inspiratory respiratory bins for subsequent comparison.

The overall image quality of 5D reconstructions was assessed in simulated data by the reconstruction error relative to the ground truth reference, calculated over a region of interest containing the heart, and across all cardiac and respiratory bins. In the absence of a ground truth reference for patient data, image quality for all 5D reconstructions was graded by three independent blinded expert reviewers (E.T., T.B., and M.P.). For each patient, mid-diastolic images were manually selected by a separate reviewer for both End-Exp and End-Ins and presented to the blinded reviewers in random order. Image grades were assigned according to the following Likert scale: 0—non-diagnostic, 1—marked blurring with limited diagnostic value, 2—moderate blurring with diagnostic value, 3—mild blurring with good diagnostic value, 4—excellent diagnostic value, with half grades assigned to images that fall between the five categories. For all quantitative metrics across the three experiments, statistically significant differences between reconstructions were measured using paired t-tests with a p-value less than 0.05 considered significant.

Additionally, 5D IIMC images were reconstructed a second time to 25 cardiac frames, reformatted into short-axis views, segmented, and compared to reference standard 2D CINE images. For both 2D and 5D images, left and right ventricular volume and ejection fraction measurements were performed using Circle cvi42 (Circle Cardiovascular Imaging, Calgary, Alberta, Canada). The measured values were compared between 2D and 5D using linear regression, Bland-Altman analysis, and Pearson’s correlation coefficient. The diagnostic image quality of 2D and 5D short-axis images was assessed and compared according to a five-point quality scale ranging from 1—insufficient due to a combination of blurring motion and insufficient contrast to 5—excellent without blurring, motion, and with excellent contrast.

Finally, multiplanar reformats of the right coronary artery (RCA), left anterior descending artery (LAD), and left circumflex artery (LCX) from three representative patients were obtained using Soap-bubble [41]. Images during End-Exp and End-Ins were manually selected during mid-diastole to provide a benchmark comparison of fine anatomical structures from image reconstructions with and without IIMC.

### 2.5 Reconstruction parameters

All fNAV reconstructions ran for five iterations of the gradient descent algorithm resulting in approximately 1 minute of reconstruction time per respiratory bin. All CS reconstructions were performed using an Alternating Direction Method of Multipliers (ADMM) algorithm [21], [42] with the augmented Lagrangian (rho) fixed to 0.06, and a total of 8 ADMM iterations. All image reconstructions were performed offline in MATLAB (MathWorks, Natick, Massachusetts) on a workstation equipped with 2 Intel Xeon CPUs (Intel, Santa Clara, California), 512 GB of RAM, and a NVIDIA Tesla GPU (Nvidia, Santa Clara, California). Estimations of intra-bin displacement using fNAV and inter-bin non-rigid motion using image registration were also performed in MATLAB. The total reconstruction time including all steps of the proposed framework was approximately 8 hours 5D dataset.

## 3 Results

### 3.1 Experiment 1: Impact of intra-bin respiratory motion correction

**Fig. 2:**
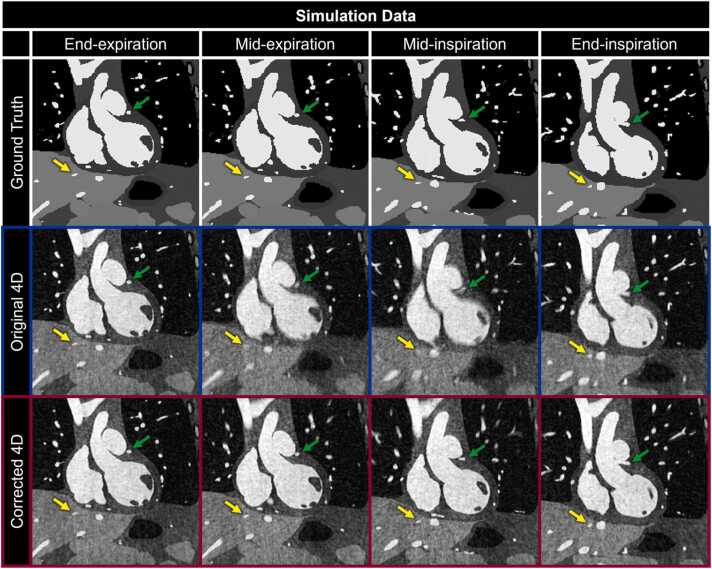
Impact of intra-bin respiratory motion *correction* on simulated data. Coronal reformats of representative respiratory motion-resolved 4D image reconstructions of simulated data are shown with (Corrected 4D) and without (Original 4D) intra-bin *correction* for four respiratory bins and compared to the simulated ground truth. Yellow (a branch of the middle hepatic vein) and green (a portion of the left anterior descending coronary artery) arrows indicate regions where uncorrected intra-bin motion degrades image quality, particularly during inspiration. 4D: four-dimensional.

**Fig. 3:**
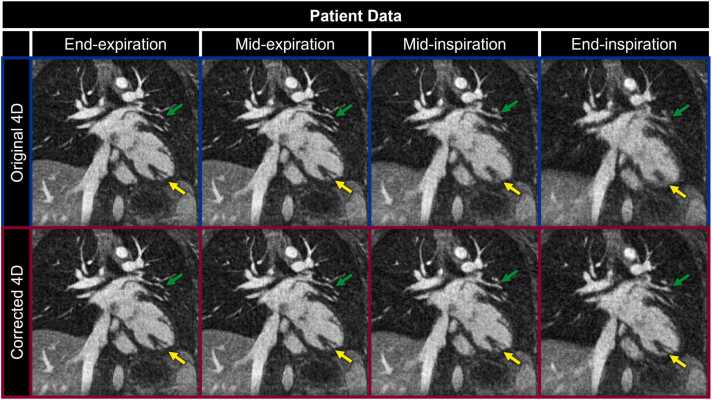
Impact of intra-bin respiratory motion *correction* on patient data. Coronal reformats of representative respiratory motion-resolved 4D image reconstructions of patient data are shown with (Corrected 4D) and without (Original 4D) intra-bin *correction* for four respiratory bins. Yellow (papillary muscle in the left ventricle) and green (branch of the pulmonary vein) arrows highlight anatomical structures that become blurrier due to uncorrected intra-bin motion. 4D: four-dimensional.

In simulated data, fNAV provided reliable estimates of intra-bin motion with an error that was smaller than the acquired spatial resolution (0.33 ± 0.12 mm). There was variability across the respiratory phases when considering the range of intra-bin motion present in both simulated (End-Exp: 0.42 ± 0.29 mm, Mid-Exp: 2.17 ± 1.08 mm, Mid-Ins: 2.31 ± 1.15 mm, End-Ins: 1.39 ± 0.64 mm) and patient (End-Exp: 0.79 ± 0.40 mm, Mid-Exp: 0.58 ± 0.28 mm, Mid-Ins: 0.73 ± 0.39 mm, End-Ins: 2.15 ± 1.81 mm) data. Visual comparison of respiratory motion-resolved 4D image reconstructions of simulated data (Fig. 2) with (Corrected 4D) and without (Original 4D) intra-bin *correction* corroborates these findings and demonstrates the clear effects of increased intra-bin motion, particularly during inspiration. Without motion *correction*, blood vessels in the liver (Fig. 2 yellow arrows) and LAD (Fig. 2 green arrows) have reduced conspicuity when compared to motion-free references (Ground Truth). Using the proposed method for intra-bin *correction*, image quality is comparable to the ground truth reference with excellent delineation of fine anatomical details despite the underlying motion. Similarly, visual comparison of in vivo image reconstructions (Fig. 3) shows a decrease in quality during mid and End-Ins for reconstructions without motion *correction* including decreased visibility of papillary muscles in the left ventricle (Fig. 3 yellow arrows) and LAD (Fig. 3 green arrows). In contrast, image quality is again maintained throughout the respiratory phases using intra-bin motion *correction*.

**Fig. 4:**
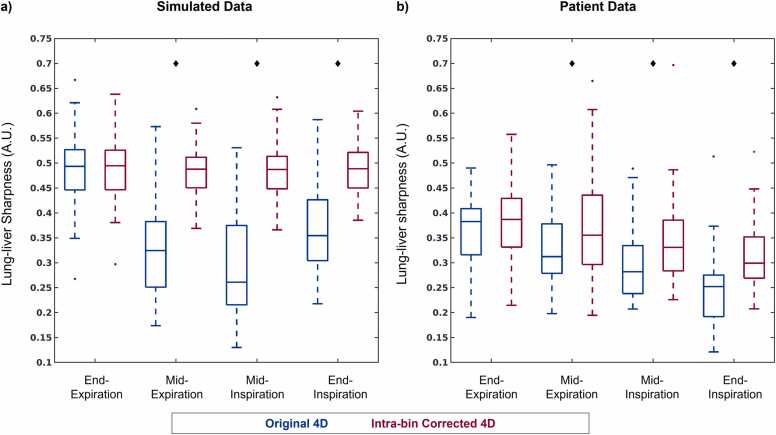
Quantitative impact of intra-bin respiratory motion *correction*. Lung-liver interface sharpness from respiratory motion-resolved 4D image reconstructions of (a) simulated (N = 50) and (b) patient (N = 32) data with (Corrected 4D) and without (Original 4D) intra-bin *correction* across four respiratory phases. The extents of the boxplots indicate the total range with the median indicated by the central mark, the 25th and 75th percentiles by the bottom and top edges, respectively, and statistically significant differences (p < 0.05) between the two reconstructions are denoted by black diamonds. 4D, four-dimensional; A.U., arbitrary units.

These qualitative results are corroborated by quantitative analysis of lung-liver interface sharpness performed for all simulated (N = 50) and patient (N = 32) data (Fig. 4). The sharpness of motion-corrected images is relatively constant across the four respiratory phases for simulated data (End-Exp: 0.49 ± 0.06, Mid-Exp: 0.48 ± 0.05, Mid-Ins: 0.48 ± 0.05, End-Ins: 0.49 ± 0.06) and patient data (End-Exp: 0.38 ± 0.08, Mid-Exp: 0.37 ± 0.11, Mid-Ins: 0.35 ± 0.10, End-Ins: 0.31 ± 0.07) but decreases for uncorrected phantom data (End-Exp: 0.49 ± 0.07, Mid-Exp: 0.33 ± 0.10, Mid-Ins: 0.29 ± 0.10, End-Ins: 0.37 ± 0.08) and uncorrected patient data (End-Exp: 0.36 ± 0.08, Mid-Exp: 0.32 ± 0.07, Mid-Ins: 0.29 ± 0.07, End-Ins: 0.25 ± 0.07). Consequently, a statistically significant improvement in the sharpness of motion-corrected images (relative to uncorrected) is observed for all respiratory phases except End-Exp for simulated data (End-Exp: p = 0.86, Mid-Exp: p < 0.001, Mid-Ins: p < 0.001, End-Ins: p < 0.001) and patient data (End-Exp: p = 0.40, Mid-Exp: p = 0.03, Mid-Ins: p = 0.01, End-Ins: p < 0.001).

### 3.2 Experiment 2: Impact of inter-bin respiratory motion compensation

Overall, the image reconstruction and registration pipeline produced accurate deformation fields. The average error in total displacement was 0.65 ± 0.18 mm with maximum errors of 3.01 mm occurring near the lower border of the image where the maximum displacement of the liver entering and exiting the imaging FOV causes some irregularities in the registration but does not affect the region of interest containing the heart.

**Fig. 5:**
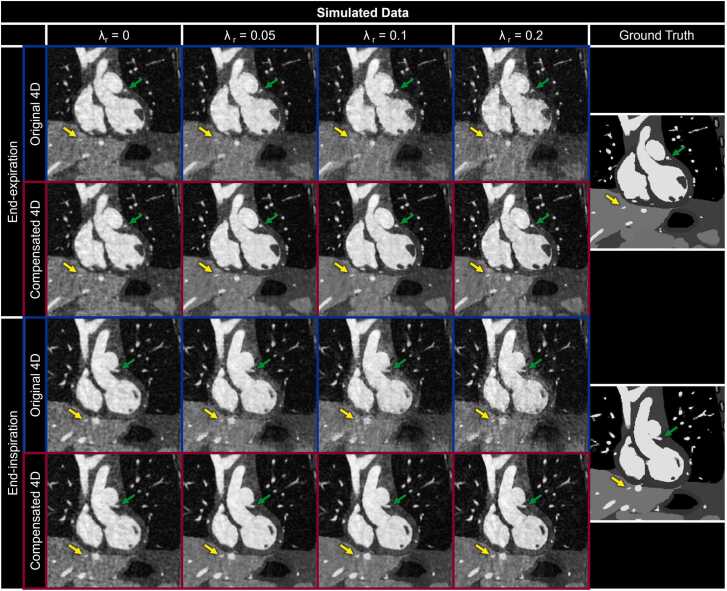
Impact of inter-bin respiratory motion compensation on simulated data. Coronal reformats of representative respiratory motion-resolved 4D image reconstructions of simulated data are shown with (Compensated 4D) and without (Original 4D) inter-bin *compensation* for two respiratory bins and compared to the simulated reference (Ground Truth) across a range of regularization weights (λ_r_). Yellow (a branch of the middle hepatic vein) and green (left anterior descending coronary artery) arrows point to regions where uncompensated inter-bin respiratory motion leads to blurrier image reconstructions with increased λ_r_. 4D: four-dimensional.

**Fig. 6:**
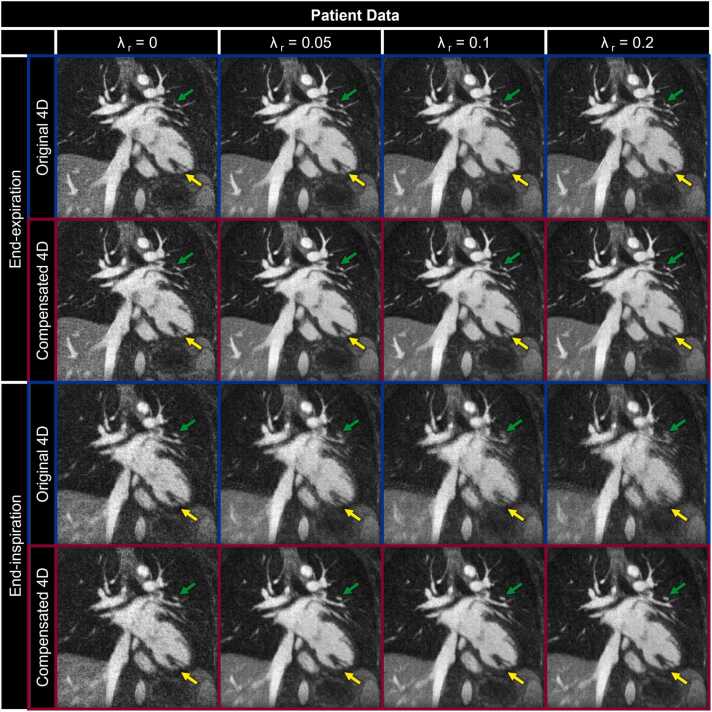
Impact of inter-bin respiratory motion compensation on patient data. Coronal reformats of representative respiratory motion-resolved 4D image reconstructions of patient data are shown with (Compensated 4D) and without (Original 4D) inter-bin *compensation* for two respiratory bins across a range of regularization weights (λ_r_). Yellow arrows (papillary muscle in the left ventricle) and green arrows (a branch of the pulmonary vein) demonstrate fine details that are lost due to uncompensated inter-bin respiratory motion for increasing λ_r_. 4D: four-dimensional.

Visual comparison of representative respiratory motion-resolved 4D intra-bin corrected image reconstructions of simulated data with (Compensated 4D) and without (Original 4D) inter-bin *compensation* (Fig. 5) shows a clear improvement in overall image quality using the proposed method for motion *compensation* for all non-zero regularization weights (λ_r_). In particular, increased regularization weight reduces the visibility of blood vessels in the liver (Fig. 5 yellow arrows) and LAD (Fig. 5 green arrows) for uncompensated reconstructions of both respiratory phases when compared to the ground truth reference. The quality of motion-compensated reconstructions is much less dependent on λ_r_ and is comparable to the ground truth across the tested range. A similar trend is observed in vivo (Fig. 6), wherein the visibility of fine features, such as papillary muscles (Fig. 6 yellow arrows) and LAD (Fig. 6 green arrows), is reduced with increasing λ_r_. However, for in vivo data, this difference is much more pronounced during End-Ins.

**Fig. 7:**
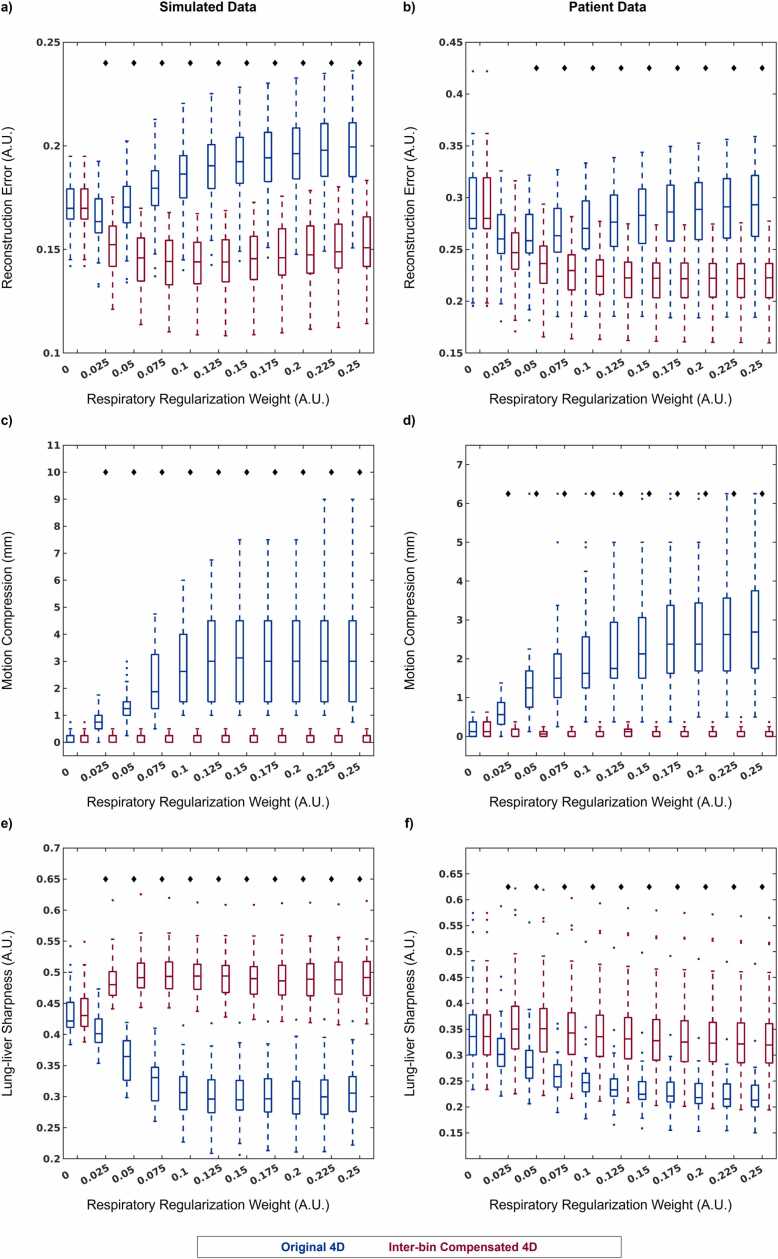
Quantitative impact of inter-bin respiratory motion *compensation*. Each metric is calculated from respiratory motion-resolved 4D image reconstructions of (left column) simulated (N = 50) and (right column) patient (N = 32) data with (Compensated 4D) and without (Original 4D) inter-bin *compensation* across a range of regularization weights (λ_r_). Reconstruction error (a and b), motion compression (c and d), and lung-liver sharpness (e and f) are presented as boxplots indicating the total range with the median indicated by the central mark, the 25th and 75th percentiles by the bottom and top edges, respectively, and statistically significant differences (p < 0.05) between the two reconstructions are denoted by black diamonds. 4D, four-dimensional; A.U., arbitrary units.

Quantitative comparison of reconstructions with and without inter-bin motion *compensation* again corroborates the qualitative comparison provided by Figs. 5 and 6 for both simulated and in vivo data, respectively. The mean reconstruction error in simulated data (Fig. 7a) across all tested λ_r_ was significantly lower (p <0.001) for inter-bin motion compensated images (0.15 ± 0.02) than for uncompensated images (0.18 ± 0.02). Likewise, for patient data (Fig. 7b) the reconstruction error is significantly (p < 0.001) lower for compensated images (0.23 ± 0.04) than for uncompensated images (0.27 ± 0.04). Overall, reconstruction error increases with λ_r_ and has a relatively sharp minimum for uncompensated images. Conversely, the reconstruction error for images with inter-bin motion *compensation* increases more gradually with significantly lower values (p < 0.05) for all non-zero λ_r_ (10/11 tested values) in simulated data and all λ_r_ greater than 0.05 for patient data (9/11 tested values).

As observed in Figs. 5 and 6, the lung-liver interface in uncompensated images appears blurred for increasing λ_r_ resulting in both a perceived and quantifiable compression of respiratory motion (Fig. 7c and d) and a decrease in the interface sharpness (Fig. 7e and f). The mean motion compression in simulated data (Fig. 7c) across all tested λ_r_ was significantly lower (p < 0.001) for inter-bin motion compensated images (0.20 ± 0.17 mm) than for uncompensated images (2.45 ± 1.80 mm). Similarly, for patient data (Fig. 7d) the motion compression is significantly (p < 0.001) lower for compensated images (0.11 ± 0.12 mm) than uncompensated images (1.99 ± 1.58 mm). The mean lung-liver interface sharpness in simulated data (Fig. 7e) across all tested λ_r_ was significantly higher (p < 0.001) for inter-bin motion compensated images (0.49 ± 0.04) than for uncompensated images (0.33 ± 0.06 mm).

Similarly, for patient data (Fig. 7f) sharpness is significantly (p < 0.001) higher for compensated images (0.35 ± 0.09) than uncompensated images (0.26 ± 0.07). Overall, image reconstructions with inter-bin *compensation* do not incur noticeable compression of the respiratory motion resulting in a flat trend for interface sharpness across λ_r_. Finally, for intra-bin corrected and inter-bin compensated image reconstructions from both simulated and patient data, motion compression was significantly (p < 0.05) lower, and lung-liver interface sharpness was significantly higher (p < 0.05) for all non-zero λ_r_ values (10/11 tested values).

### 3.3 Experiment 3: Evaluating intra-bin corrected inter-bin compensated 5D whole-heart MRI

**Fig. 8:**
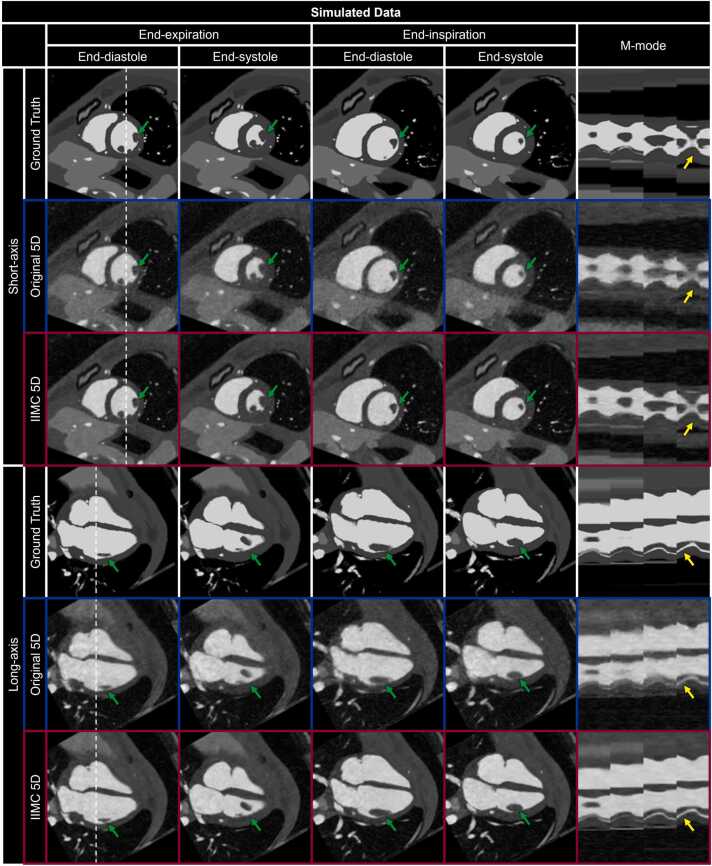
Intra-bin corrected inter-bin compensated 5D reconstructions of simulated data. Short-axis and long-axis reformats of representative cardiac and respiratory motion-resolved 5D image reconstructions of simulated data are shown with (IIMC 5D) and without (Original 5D) the proposed combination of intra-bin *correction* and inter-bin *compensation* of respiratory motion. The ground truth simulated images are provided for reference. For each reconstruction and reformatted view, end-diastolic and end-systolic cardiac phases are shown during End-Exp and End-Ins, as well as an m-mode representation of the region along the dashed white lines showing the temporal evolution of signal across cardiac and respiratory phases. Green (papillary muscle in the left ventricle) and yellow (blood-myocardium interface) arrows indicate regions where respiratory motion can degrade image quality if not accounted for. An animated version of this figure is included in Additional file 1. 5D, five-dimensional; IIMC, intra-acquisition correction and inter-acquisition compensation of respiratory motion.

**Fig. 9:**
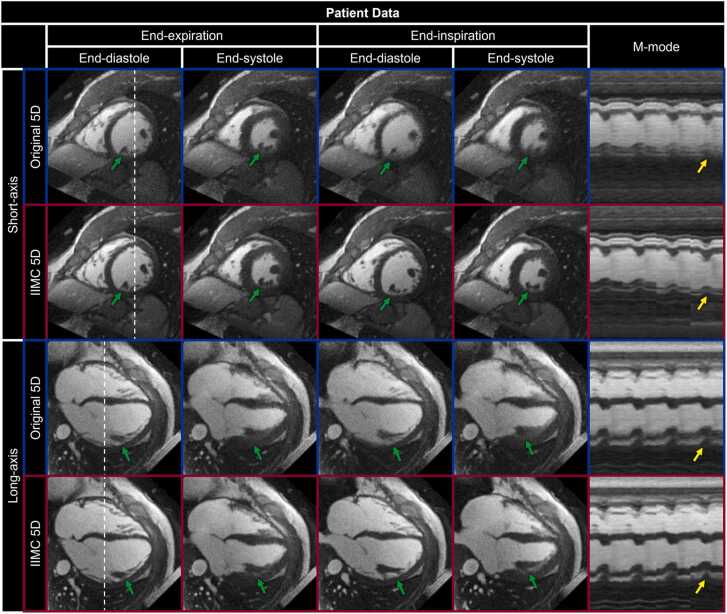
Intra-bin corrected inter-bin compensated 5D reconstructions of patient data. Short-axis and long-axis reformats of representative cardiac and respiratory motion-resolved 5D image reconstructions of patient data are shown with (IIMC 5D) and without (Original 5D) the proposed combination of intra-bin *correction* and inter-bin *compensation* of respiratory motion. For each reconstruction and reformatted view, end-diastolic and end-systolic cardiac phases are shown during End-Exp and End-Ins, as well as an m-mode representation of the region along the dashed white lines showing the temporal evolution of signal across cardiac and respiratory phases. Green (papillary muscles in the left ventricle) and yellow (blood-myocardium interface) arrows indicate regions where respiratory motion can degrade image quality if not accounted for. An animated version of this figure is included in Additional file 2. 5D, five-dimensional; IIMC, intra-acquisition correction and inter-acquisition compensation of respiratory motion.

Cardiac and respiratory motion-resolved 5D image reconstructions of both simulated and patient data were performed using the regularization weights (λ_r_) that minimized the reconstruction error as determined by experiment 2 for reconstructions with (λ_r_ = 0.1) and without (λ_r_ = 0.01) the proposed combination of intra-bin *correction* and inter-bin *compensation* of respiratory motion. 5D image reconstructions (Fig. 8) of simulated data using intra-bin *correction* and inter-bin *compensation* of respiratory motion (IIMC 5D) demonstrate excellent image quality that is comparable to the simulated reference (Ground Truth) and significantly improves on the previously published reconstruction framework (Original 5D). Both radial and longitudinal contractions of the ventricles are well visualized in the short-axis and long-axis reformats, respectively, when comparing the end-diastolic and end-systolic cardiac phases. However, the visibility of papillary muscles and blood-myocardium interface is reduced in the Original 5D reconstructions for both End-Exp and End-Ins but is preserved using IIMC 5D. Similarly, IIMC 5D reconstructions of patient data (Fig. 9) provide a clear depiction of ventricular contraction with fine anatomical details well preserved even during End-Ins whereas residual motion reduces perceived sharpness in the Original 5D reconstructions.

**Fig. 10:**
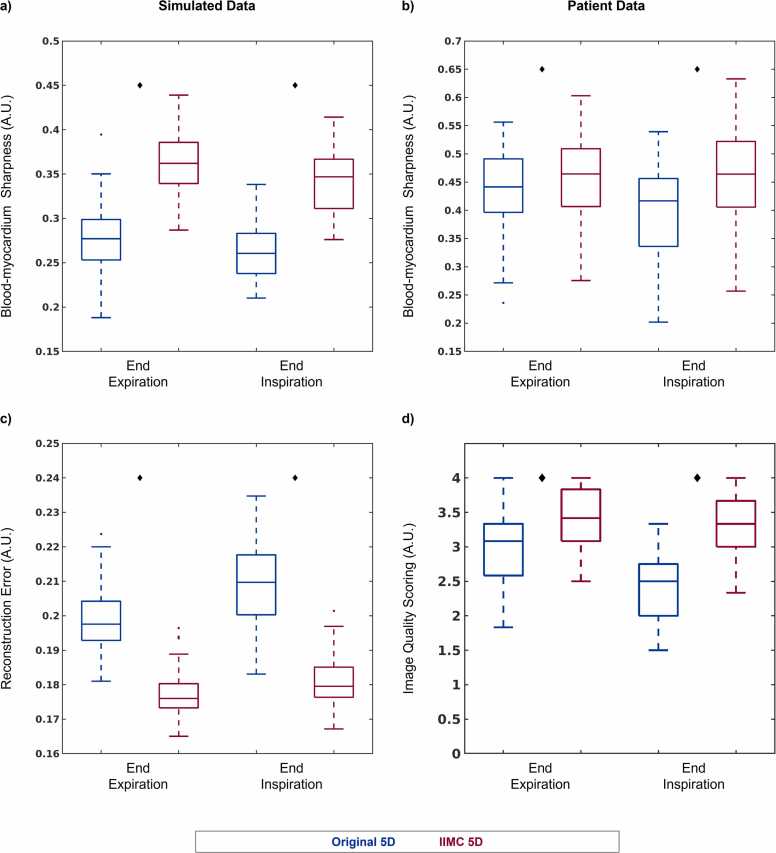
Quantitative evaluation of intra-bin corrected inter-bin compensated 5D whole-heart CMR. Each metric is calculated from cardiac and respiratory motion-resolved 5D image reconstructions of (left column) simulated (N = 50) and (right column) patient (N = 32) data with (IIMC 5D) and without (Original 5D) the proposed combination of intra-bin *correction* and inter-bin *compensation* of respiratory motion for two respiratory phases. Sharpness of the blood-myocardium interface (a and b), reconstruction error (c), and consensus image quality scoring from expert reviewers (d) are presented as boxplots indicating the total range with the median indicated by the central mark, the 25th and 75th percentiles by the bottom and top edges, respectively, and statistically significant differences (p < 0.05) between the two reconstructions are denoted by black diamonds. 5D, five-dimensional: IIMC, intra-acquisition correction and inter-acquisition compensation of respiratory motion; CMR, cardiovascular magnetic resonance.

Qualitative evaluation of simulated and patient data is again corroborated by quantitative metrics (Fig. 10). The sharpness of the blood-myocardium interface in simulated (Fig. 10a) image reconstructions is significantly (p < 0.001) improved using IIMC 5D (End-Exp: 0.36 ± 0.04, End-Ins: 0.34 ± 0.04) relative to the Original 5D implementation (End-Exp: 0.28 ± 0.04, End-Ins: 0.26 ± 0.03). The sharpness of patient images (Fig. 10b) is also significantly (p < 0.001) improved using IIMC 5D (End-Exp: 0.45 ± 0.09, End-Ins: 0.46 ± 0.10) relative to the Original 5D implementation (End-Exp: 0.43 ± 0.08, End-Ins: 0.39 ± 0.09). Albeit, for patient data, the difference between reconstruction methods is larger for the End-Ins images than for End-Exp which agrees with the visual differences observed in Fig. 9.

Reconstruction error (Fig. 10c), which provides a metric for image quality relative to ground truth for simulated data, is significantly (p < 0.001) lower using IIMC 5D (End-Exp: 0.18 ± 0.01, End-Ins: 0.18 ± 0.01) relative to the Original 5D implementation (End-Exp: 0.20 ± 0.01, End-Ins: 0.21 ± 0.01). In the absence of a ground truth comparison for patient data, image scoring by three expert reviewers (Fig. 10d) yields significantly higher (p < 0.001) overall scores using IIMC 5D (End-Exp: 3.39 ± 0.45, End-Ins: 3.32 ± 0.45) relative to the Original 5D implementation (End-Exp: 3.02 ± 0.54, End-Ins: 2.45 ± 0.52). Once again, a larger difference is observed during End-Ins.

**Fig. 11:**
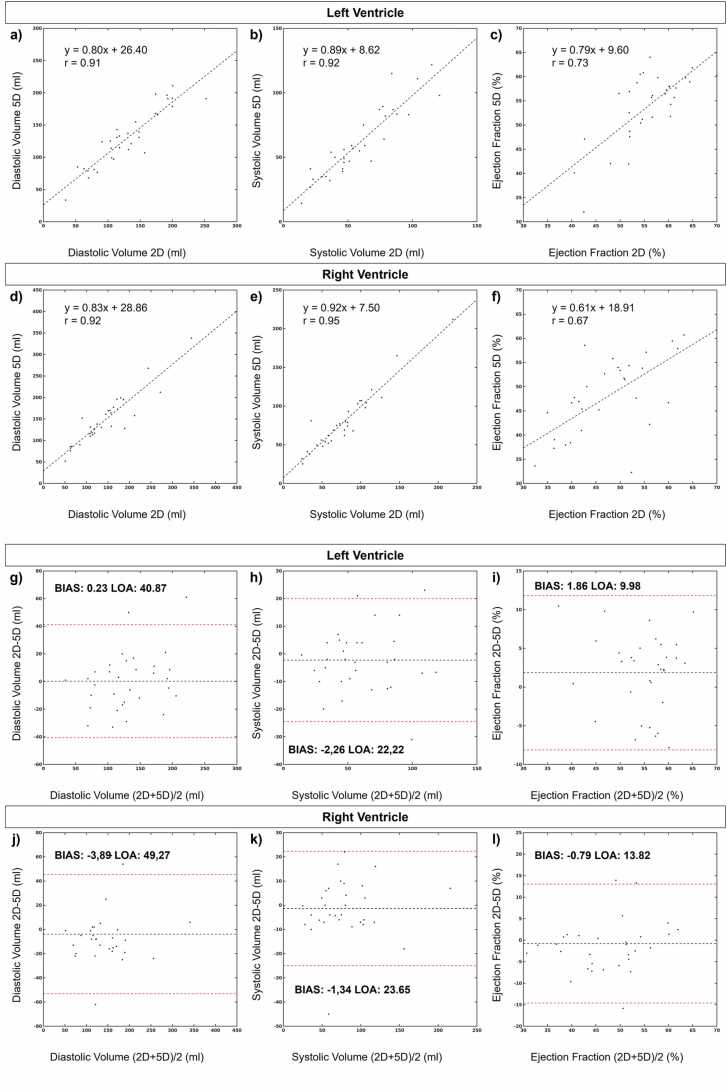
Quantitative comparison of ventricular volumes measured from 2D CINE and intra-bin corrected inter-bin compensated 5D whole-heart CMR. 2D, two-dimensional; 5D, five-dimensional; LOA, limits of agreement; CMR, cardiovascular magnetic resonance

Left (LV) and right ventricular (RV)segmentations were successful for all 32 patients using both the proposed IIMC 5D image reconstruction and reference 2D CINEs allowing for quantitative comparison between the two methods (Fig. 11). There was very strong correlation between 2D and 5D measurements of LV diastolic volume (r: 0.91), LV systolic volume (r: 0.92), RV diastolic volume (r: 0.92), and RV systolic volume (r: 0.95). There was a strong correlation between 2D and 5D measurements of LV (r: 0.73) and RV (r: 0.67) ejection fraction. Overall, there was low bias when comparing 2D and 5D measurements through Bland-Altman analysis (Fig. 11) but relatively large limits of agreement. This may be attributed to slightly lower image quality scores for 2D images (3.75 ± 0.80) relative to 5D images (4.59 ± 0.66, p < 0.001), wherein the 2D images with the lowest scores contained artifact likely from spurious ECG triggers.

**Fig. 12:**
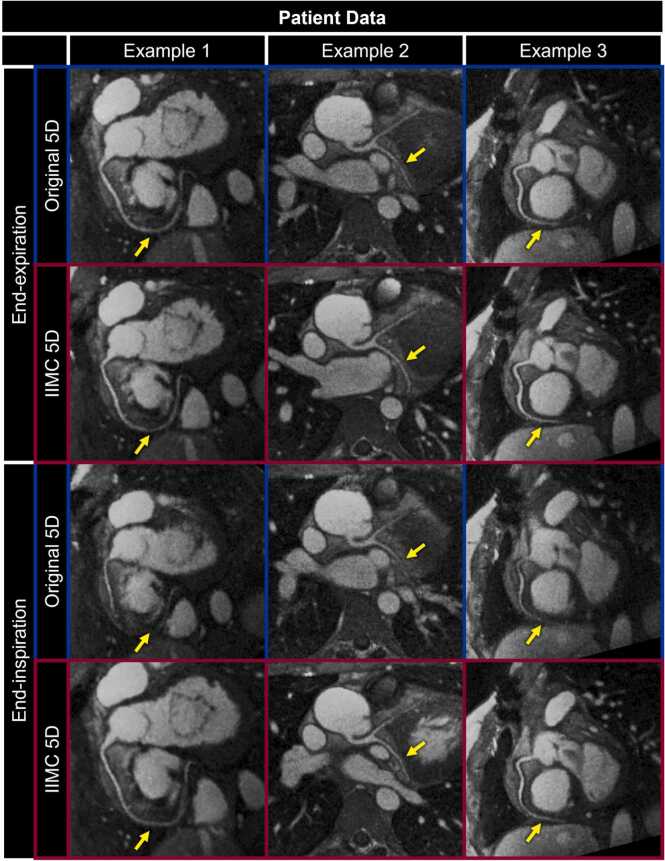
Coronary magnetic resonance angiography using intra-bin corrected inter-bin compensated 5D whole-heart CMR. Multiplanar reformats of the RCA (examples 1 and 3) and LAD+LCX (example 2) are shown during End-Exp and End-Ins for image reconstructions of patient data with (IIMC 5D) and without (Original 5D) the proposed combination of intra-bin *correction* and inter-bin *compensation* of respiratory motion. Yellow arrows indicate regions where vessel conspicuity is clearly improved using IIMC 5D, particularly during End-Ins. 5D, five-dimensional; IIMC, intra-acquisition correction and inter-acquisition compensation of respiratory motion; CMR, cardiovascular magnetic resonance.

Finally, multiplanar reformats of coronary arteries from three example patient datasets provide a benchmark comparison of the proposed IIMC 5D image reconstruction framework to the Original 5D approach (Fig. 12). Vessel conspicuity is similar between reconstructed images during End-Exp but is significantly improved during End-Ins using IIMC 5D. In fact, the overall quality of multiplanar reformats is comparable whether performed during End-Exp or End-Ins, consistent with the qualitative and quantitative results shown in Fig. 7, Fig. 8, Fig. 9.

## 4 Discussion

In this work, we developed and validated a novel framework for intra-bin *correction* and inter-bin *compensation* of respiratory motion in free-running 5D whole-heart CMR. We successfully tested our three hypotheses showing that 1) correcting intra-bin motion using fNAV improves image sharpness, 2) the inclusion of non-rigid deformation fields in the CS reconstruction enables larger regularization weights to reduce artifact without incurring motion blur, and 3) the combined approach for intra-bin *correction* and inter-bin *compensation* of respiratory motion results in improved image quality for 5D whole-heart MRI across the reconstructed respiratory bins. Systematic and comprehensive validation of the proposed framework was performed using a complex numerical simulation. Finally, multiple qualitative and quantitative comparisons were made between simulated data and the known ground truth reference and then corroborated by reconstructions of 32 patient datasets demonstrating the robustness of our approach.

The proposed reconstruction framework does not require specific hardware or sequence modifications and is therefore readily applicable to free-running 3D radial acquisitions beyond the gradient echo sequence used in this work, including previously published bSSFP [20], [43], natively fat suppressed [44], and flow-sensitive [18] variants. Furthermore, this method could be extended to other k-space trajectories [45] provided the data can be retrospectively and arbitrarily binned and a respiratory signal can be obtained, the latter of which could come from the sequence [46] or additional hardware [47].

This study presents, to our knowledge, the first investigation of the impact of intra-bin motion in 5D whole-heart MRI. Our results clearly show that image quality, particularly during inspiration, is diminished due to intra-bin motion using four respiratory bins as previously published [15], [20], [21]. While an increase in the number of bins may improve the quality of the inspiration phases by reducing the variability of the residual respiratory motion, it would also increase the level of undersampling whereas the proposed use of fNAV to correct the intra-bin motion is able to provide consistent quality throughout the respiratory cycle. Nevertheless, there is unexplored potential in using respiratory motion-resolved imaging to study the effects of respiration on cardiac output and blood flow, and therefore the current work provides a necessary solution that may decouple the effects of image quality from the effects of physiology. Furthermore, the current gold standard techniques for cardiac function and blood flow measurements are 2D CINE bSSFP and 2D phase-contrast CMR, respectively. Both of these techniques are regularly performed under-breath hold wherein End-Exp tends to be more reproducible in terms of diaphragm position, but End-Ins may be better tolerated by patients. This leads to varied practices from center to center [48]. Therefore, the ability to reliability reconstruct free-running datasets during End-Exp or End-Ins could be a valuable asset when comparing and validating against 2D methods.

Our results also demonstrate that using inter-bin *compensation* of respiratory motion, higher regularization weights can be chosen which reduces undersampling artifact and improves overall image quality without inducing blur due to uncompensated motion. These improvements could be further leveraged to improve the acquired spatial resolution, the reconstructed cardiac temporal resolution, the number of reconstructed respiratory bins or to shorten scan times. In particular, by enabling the reconstruction of more respiratory bins, the impact of intra-bin motion may be further reduced. Furthermore, the interplay between image quality and user-defined parameters is well characterized [49] and may impact the wider clinical adoption of such techniques given the possibility of “over-regularizing” and creating image artifacts in the reconstruction process. Our results show a relatively broad optimum for multiple quality metrics, including reconstruction error, motion compression, and sharpness (Fig. 7). As a result, the final image reconstruction is less sensitive to the choice of user-defined regularization weights in respiratory-resolved imaging, with relatively minor changes in overall image quality across the tested weights using IIMC 5D compared to the Original 5D implementation. This decreased sensitivity to regularization weights may facilitate clinical adoption by providing a more reproducible reconstruction.

When comparing ejection fraction measurements between our proposed IIMC 5D reconstruction to reference standard 2D imaging, we observed a low bias but with large limits of agreement. While this may be attributed to differences in image quality, we in fact observed overall higher image quality for the 5D images and noted some artifacts possibly due to spurious ECG triggering in the lowest-scored 2D CINE images. Further comparison in a larger cohort with consistent 2D CINE imaging is therefore warranted to further validate IIMC 5D ejection fraction measurements. Additionally, the effects of temporal regularization along the cardiac dimension may also play a role in this discrepancy and therefore need to be further studied.

In general, we did not explore the effects of the cardiac regularization weights in this work and in fact kept them constant to provide a fair comparison between reconstructions with and without correction/compensation. As a result, some of the gains or shortcomings of the current framework in terms of overall image quality may be biased by the cardiac regularization weight.

While beyond the scope of this work, a natural extension of the proposed method for inter-bin motion *compensation* is to estimate and apply nonrigid deformation fields also to adjacent cardiac phases thus further reducing the dependency on user-defined regularization weights [23], [50]. This may yield even greater improvement in the resulting 5D images providing further means for higher resolution of abbreviated scan times. Nevertheless, the estimation of nonrigid deformation fields requires initial images which in our framework consisted of a computational efficient gridded reconstruction of the respiratory motion-resolved data. For cardiac motion-resolved data, which are inherently more under sampled, the gridded reconstruction more not suffice for accurate estimation of deformation fields and therefore a computationally demanding initial reconstruction may be required.

## 5 Limitations

The patient cohort included in this study was injected with a contrast agent (ferumoxytol) prior to the MRI exam as part of the clinical protocol at our institution. Ferumoxytol provides a significant T1-shortening effect that combined with a gradient echo acquisition provides excellent blood-myocardium contrast. Despite the advantages of increased signal levels for overall image quality and achievable acceleration factors, the use of contrast agents, particularly in pediatric populations may vary significantly among institutions, including the availability of ferumoxytol. Nevertheless, the individual components of the current work, including fNAV, nonrigid motion *compensation*, and multi-dimensional CS, have each been previously described for acquisitions with native contrast. Nevertheless, additional validation work is warranted to demonstrate the robustness of the proposed framework using free-running acquisitions without ferumoxytol [20], [43], [44]. This should include comparisons to other motion-compensated strategies without the use of contrast agents [51] and further comparison to native contrast 2D CINE imaging which benefits from inflow effects.

Simulation and patient results clearly demonstrate the value of correcting intra-bin respiratory motion. However, the translational correction applied to k-space, while sharpening features of interest (i.e., the heart) comes at the expense of blurring structures such as the spine and chest wall that are either static or undergoing a different motion pattern. In this work, we found that using four respiratory bins provided an acceptable amount of intra-bin motion that could be corrected without creating significant artifact in static structures. An alternative would be to reconstruct more respiratory bins albeit at the cost of a higher degree of undersampling or requiring long acquisitions.

Similarly, simulation and patient results clearly demonstrate the value of including motion fields in the CS reconstruction to *compensate* for inter-bin motion. Nevertheless, with sufficient high regularization weights, it is possible that errors in the motion field estimate may propagate and induce non-physiological motion leading to artifact. While the numerical simulation provides some evidence that the current estimation of non-rigid motion is accurate, further validation in programmable physical moving phantoms may be useful. Additionally, estimation of motion fields could be integrated into each iteration of the CS algorithm which may yield further improvements.

Validation of our proposed framework was largely performed using a numerical simulation whose results were then corroborated by in vivo data. The goal of the simulation was to provide controllable variability in the amplitude and frequency of respiratory motion and a known ground truth. Still, as with any numerical simulation, a number of factors limit the overall realism of the resulting data and images. For example, the cardiac anatomy is representative of a “normal” heart whereas in vivo data came from a cohort of CHD patients. Additionally, the simulated CMR physics consisted of a simplified analytical signal equation and lacked common sources of artifact, such as field inhomogeneities, signal dephasing due to flowing blood, and realistic noise. As a result, there were some small differences in the simulation and patient results with a less dramatic improvement in image quality observed for the end-expiratory images from patient data. This is likely due to differences between the simulated respiratory curves and their in vivo counterparts. This could be further tested by using the respiratory curves from patient data as input to the simulation framework but is beyond the scope of the current study and would not likely change the overall analysis.

## 6 Conclusion

Intra-bin *correction* and inter-bin *compensation* of respiratory motion significantly improve the quality of cardiac and respiratory motion-resolved 5D whole-heart MRI with a decreased dependence on user-defined reconstruction parameters. These improvements may be exploited for higher resolution or abbreviated scan times. With further study of diagnostic impact and comparison to gold standards, this may facilitate greater clinical adoption of respiratory-resolved whole-heart imaging while working toward a deeper exploration of respiratory-driven changes in physiological measurements of interest.

## Funding

Matthias Stuber is the principal investigator on the Swiss National Science Foundation Grants 320030_173129 and 201292 that funded part of this research. Christopher Roy is the principal investigator on Swiss National Science Foundation Grant PZ00P3_202140 that funded part of this research.

## Author contributions

**Tobias Rutz:** Writing – review and editing, Formal analysis, Data curation. **Estelle Tenisch:** Writing – review and editing, Formal analysis, Data curation, Conceptualization. **Aurélien Bustin:** Writing – review and editing, Software, Methodology. **Ludovica Romanin:** Writing – review and editing, Data curation. **Salim Si-Mohamed:** Writing – review and editing, Resources, Formal analysis, Data curation. **Jérôme Yerly:** Writing – review and editing, Methodology, Formal analysis. **Bastien Milani:** Writing – review and editing, Software, Methodology. **Christopher W. Roy:** Writing – review and editing, Writing – original draft, Validation, Methodology, Investigation, Funding acquisition, Formal analysis, Data curation, Conceptualization. **Matthias Stuber:** Writing – review and editing, Writing – original draft, Supervision, Resources, Project administration, Methodology, Formal analysis, Conceptualization. **Milan Prsa:** Writing – review and editing, Formal analysis, Data curation.

## Ethics approval and consent

All subjects or their legal guardian in the case of minors provided informed written consent, including permission to publish anonymized data, as part of a study approved by the local ethics review board (CER-VD 2022-01521).

## Declaration of competing interests

Ludovica Romanin’s PhD studies are supported financially by Siemens Healthcare (Erlangen, Germany). Matthias Stuber receives non-monetary research support from Siemens Healthcare (Erlangen, Germany).

## Data Availability

The datasets and algorithms used and analyzed during the current study are available from the corresponding author upon reasonable request.

## Appendix A Supplementary material

**Additional file 1: Fig. S1.** Intra-bin corrected inter-bin compensated 5D reconstructions of simulated data. Animated short-axis and long-axis reformats of representative cardiac and respiratory motion-resolved 5D image reconstructions of simulated data are shown with (IIMC 5D) and without (Original 5D) the proposed combination of intra-bin *correction* and inter-bin *compensation* of respiratory motion. The ground truth simulated images are provided for reference. For each reconstruction and reformatted view, a full cardiac cycle is shown during End-Exp and End-Ins.

.

**Additional file 2: Fig. S2.** Intra-bin corrected inter-bin compensated 5D reconstructions of patient data. Animated short-axis and long-axis reformats of representative cardiac and respiratory motion-resolved 5D image reconstructions of patient data are shown with (IIMC 5D) and without (Original 5D) the proposed combination of intra-bin *correction* and inter-bin *compensation* of respiratory motion. For each reconstruction and reformatted view, a full cardiac cycle is shown during End-Exp and End-Ins.
