# Supplementary figures and images for: Intra-bin correction and inter-bin compensation of respiratory motion in free-running five-dimensional whole-heart magnetic resonance imaging
Source: J Cardiovasc Magn Reson. 2024 Mar 16;26(1):101037. doi: 10.1016/j.jocmr.2024.101037 (PMC10987330; doi:10.1016/j.jocmr.2024.101037)

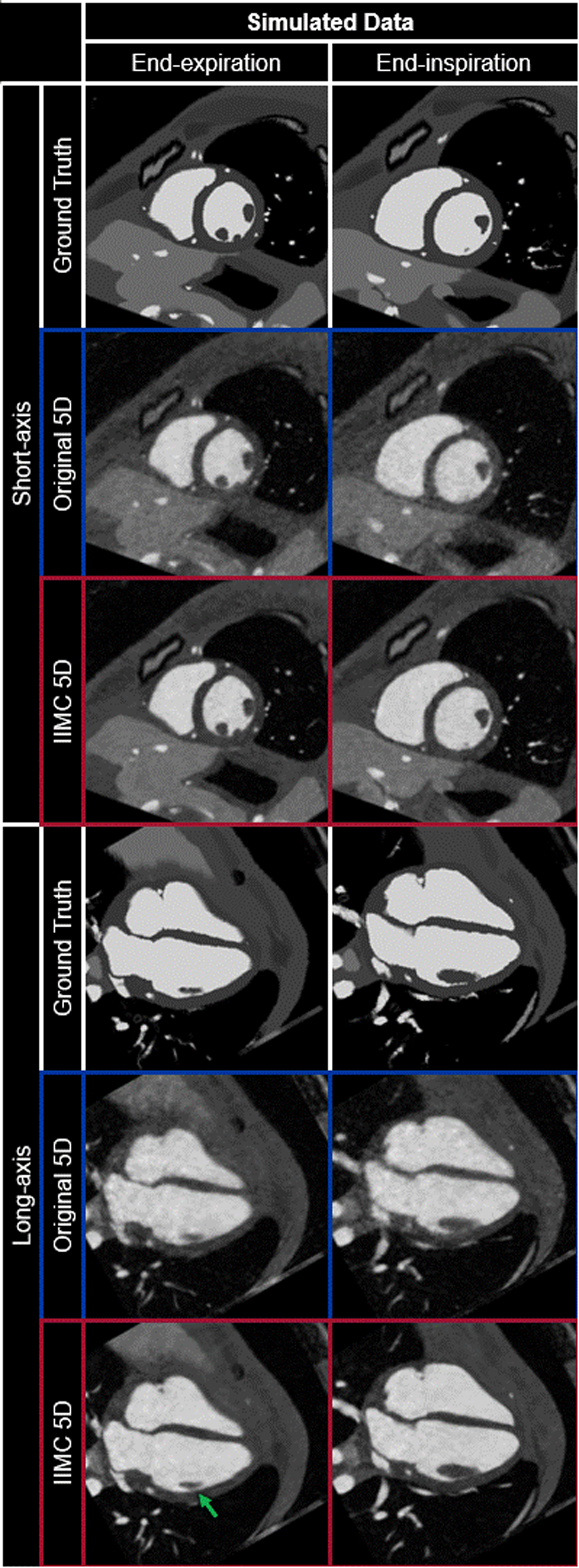

Supplement: Supplementary file 1 — Additional file 1: Fig. S1. Intra-bin corrected inter-bin compensated 5D reconstructions of simulated data. Animated short-axis and long-axis reformats of representative cardiac and respiratory motion-resolved 5D image reconstructions of simulated data are shown with (IIMC 5D) and without (Original 5D) the proposed combination of intra-bin correction and inter-bin compensation of respiratory motion. The ground truth simulated images are provided for reference. For each reconstruction and reformatted view, a full cardiac cycle is shown during End-Exp and End-Ins. [file mmc1.jpg]

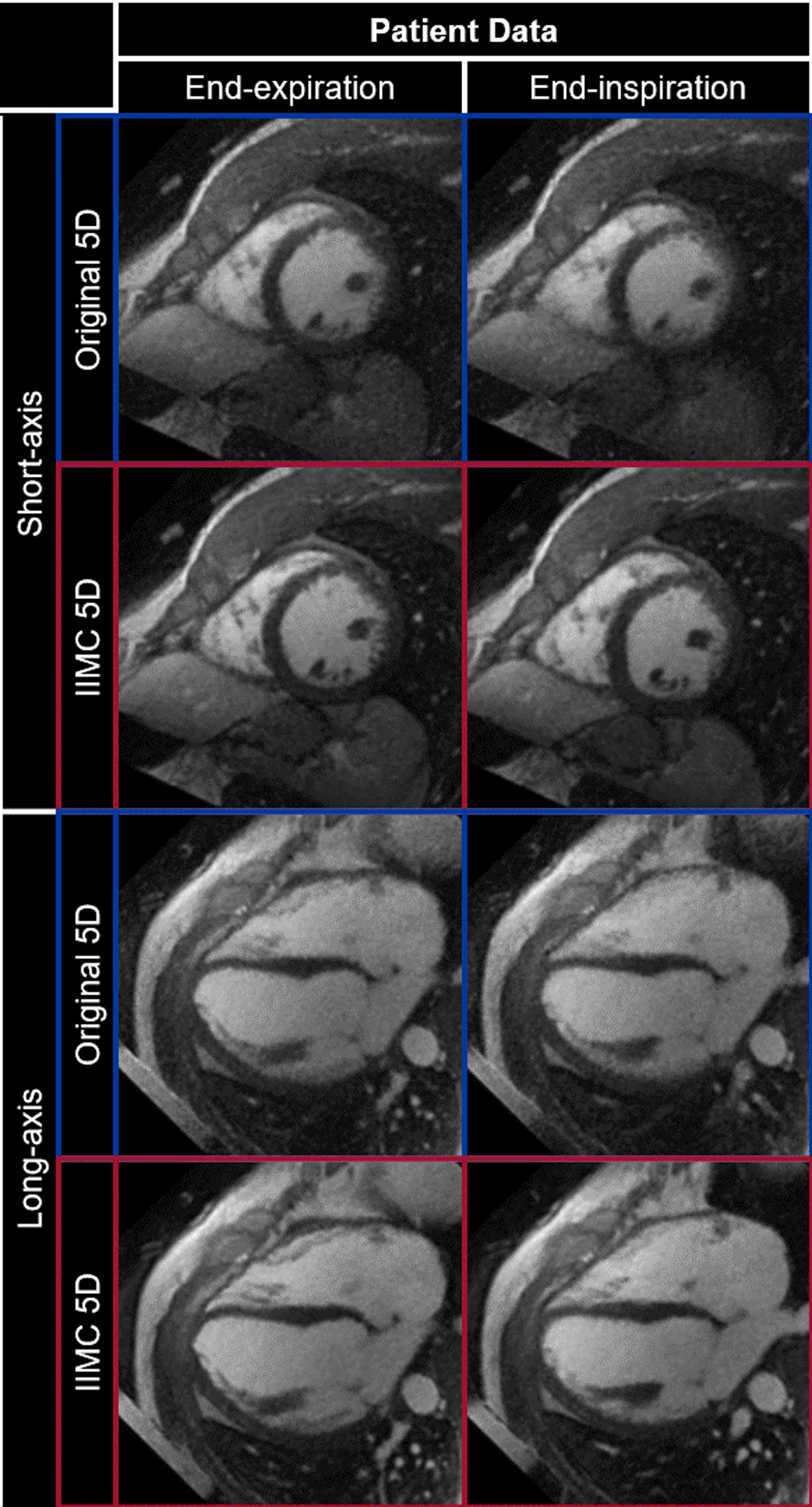

Supplement: Supplementary file 2 — Additional file 2: Fig. S2. Intra-bin corrected inter-bin compensated 5D reconstructions of patient data. Animated short-axis and long-axis reformats of representative cardiac and respiratory motion-resolved 5D image reconstructions of patient data are shown with (IIMC 5D) and without (Original 5D) the proposed combination of intra-bin correction and inter-bin compensation of respiratory motion. For each reconstruction and reformatted view, a full cardiac cycle is shown during End-Exp and End-Ins. [file mmc2.jpg]
